# Systematic review and meta‐analysis of endorectal advancement flap and ligation of the intersphincteric fistula tract for cryptoglandular and Crohn's high perianal fistulas

**DOI:** 10.1002/bjs5.50129

**Published:** 2019-01-21

**Authors:** M. E. Stellingwerf, E. M. van Praag, P. J. Tozer, W. A. Bemelman, C. J. Buskens

**Affiliations:** ^1^ Department of Surgery Amsterdam UMC, University of Amsterdam Amsterdam the Netherlands; ^2^ Robin Phillips Fistula Research Unit, St Mark's Hospital and Academic Institute Harrow HA1 3UJ UK

## Abstract

**Background:**

High perianal fistulas require sphincter‐preserving surgery because of the risk of faecal incontinence. The ligation of the intersphincteric fistula tract (LIFT) procedure preserves anal sphincter function and is an alternative to the endorectal advancement flap (AF). The aim of this study was to evaluate outcomes of these procedures in patients with cryptoglandular and Crohn's perianal fistulas.

**Methods:**

A systematic literature search was performed using MEDLINE, Embase and the Cochrane Library. All RCTs, cohort studies and case series (more than 5 patients) describing one or both techniques were included. Main outcomes were overall success rate, recurrence and incontinence following either technique. A proportional meta‐analysis was performed using a random‐effects model.

**Results:**

Some 30 studies comprising 1295 patients were included (AF, 797; LIFT, 498). For cryptoglandular fistula (1098 patients), there was no significant difference between AF and LIFT for weighted overall success (74·6 (95 per cent c.i. 65·6 to 83·7) *versus* 69·1 (53·9 to 84·3) per cent respectively) and recurrence (25·6 (4·7 to 46·4) *versus* 21·9 (14·8 to 29·0) per cent) rates. For Crohn's perianal fistula (64 patients), no significant differences were observed between AF and LIFT for overall success rate (61 (45 to 76) *versus* 53 per cent respectively), but data on recurrence were limited. Incontinence rates were significantly higher after AF compared with LIFT (7·8 (3·3 to 12·4) *versus* 1·6 (0·4 to 2·8) per cent).

**Conclusion:**

Overall success and recurrence rates were not significantly different between the AF and LIFT procedure, but continence was better preserved after LIFT.

## Introduction

Perianal fistulas are a common medical and surgical problem, resulting in an abnormal tract between the anorectal canal and the perianal skin. The estimated incidence of perianal fistula in Europe is 1·2–2·8 per 10 000 population, with a peak incidence between the age of 20 and 40 years^1^ ^2^. Despite various treatment options, many patients experience incapacitating problems with a negative impact on quality of life. Pain, discharge and recurrent abscess formation are common complaints, and in some patients sphincter and perianal tissue destruction occurs. Treatment of perianal fistula depends on clinical presentation, the underlying pathology and involvement of the external sphincter complex. Low perianal fistulas crossing less than the lower one‐third of the external anal sphincter are easily and often successfully treated by fistulotomy. High perianal fistulas are more difficult to eradicate and require sphincter‐preserving surgery because of a serious risk of incontinence.

The first step in the treatment of high perianal fistula is the insertion of a non‐cutting seton to prevent recurrent abscess formation^3^ ^4^. Subsequent closure of a high perianal fistula can be achieved by several surgical strategies, of which the endorectal advancement flap (AF) is best established^5^. During this procedure, the internal fistula opening is covered with a flap of mucosal tissue with or without muscle fibres of the internal sphincter. Possible complications are recurrence, incontinence and, although not common, necrosis of the mucosal flap. The ligation of the intersphincteric fistula tract (LIFT) procedure is an alternative to the AF, and is suggested to preserve anal sphincter function better^6^. During this procedure, the fistula tract is identified in the intersphincteric space and ligated close to the internal and external sphincters. Early reports on the LIFT procedure showed promising results with low recurrence and incontinence rates^7^.

Previous studies have tried to assess which treatment is best for high perianal fistulas. The only two RCTs^8^ ^9^ showed comparable results without significant differences in overall success and recurrence rates (around 70 and 20 per cent respectively). Differences in postoperative faecal incontinence, however, are unknown. Furthermore, there has been a lack of attention to the difference in underlying pathologies. Over 90 per cent of the perianal fistulas originate from cryptoglandular sepsis^10^, but perianal fistulas are also a major problem in patients with Crohn's disease. Up to one‐third of patients with Crohn's disease will have one or more perianal fistulas 20 years after diagnosis^11^. Crohn's perianal fistulas have a different aetiology, and are more often refractory to surgery. The risk of incontinence is greater in Crohn's disease, owing to the increased risk of diarrhoea throughout the patient's life. Surgical closure can be attempted only in patients without proctitis, and a multidisciplinary approach is required, as it has been demonstrated that results will improve under optimal levels of biological therapy.

Outcomes of the surgical closure techniques should be investigated separately for cryptoglandular and Crohn's fistulas. The aim of this study was to evaluate both procedures for either indication in terms of overall success, recurrence and incontinence rates.

## Methods

A systematic review and meta‐analysis was performed according to the PRISMA guidelines^12^.

### Search strategy

MEDLINE (PubMed), Embase (Ovid) and the Cochrane Library were searched systematically up to 13 September 2017 with the assistance of a clinical librarian. Medical subject headings (MeSH) and free‐text terms used included ‘ligation’, ‘ligation of intersphincteric fistula tract’, ‘LIFT’, ‘advancement’, ‘rectal fistula’, ‘anal fistula’, ‘anorectal fistula’, ‘perianal fistula’ and ‘fistula‐*in‐ano*’. The search was limited to studies published in the English language. There were no restrictions considering the publication date, and no other methodological filters were applied. Further details of the search terms are provided in *Appendix* S1 ( supporting information).

### Study selection

All RCTs, cohort studies and case series (more than 5 patients) describing the AF or LIFT procedure in patients with high perianal fistula of cryptoglandular and/or Crohn's origin were included. A perianal fistula was considered to be high if the tract was too high to lay open (mostly positioned in the upper two‐thirds of the external sphincter with an increased risk of incontinence). Studies not reporting success or recurrence rates and length of follow‐up, animal studies, reviews, case reports and letters were excluded. Other exclusion criteria were patients aged less than 18 years, those with a stoma, low perianal fistula, rectovaginal and rectourethral fistulas, fistulas without an external opening, and fistulas due to other aetiologies such as malignancy, trauma or human immunodeficiency virus infection. Combinations of techniques or other variants of the techniques, such as anocutaneous flaps, core‐out fistulectomy or dissection of the tract before the actual procedure, were excluded.

Two reviewers separately screened the titles and abstracts of the retrieved articles and independently assessed the full text of the remaining articles. Disagreements concerning the selection were resolved by joint discussion and, when necessary, the opinion of a third researcher was obtained. In case of overlapping study cohorts, the most informative article was chosen. Reference lists of included articles were cross‐checked to see whether any other studies could be added.

### Data collection

Data were extracted independently by two reviewers, and study authors were contacted for missing data. The composite primary outcomes of this study were overall success and recurrence rates. Overall success was defined as no presence of a persisting or recurrent fistula after a minimum follow‐up of 3 months. Overall success rates were extracted directly from the results, or calculated by subtracting recurrences from the primary success rate or by subtracting the number of persistent and recurrent fistulas from the total of included fistulas. A procedure was considered successful if the external fistula opening was closed without drainage. Recurrence rate was defined as reopening or drainage after apparent healing of the external opening, with discrimination between transsphincteric and intersphincteric recurrences after the LIFT procedure. The secondary outcome was faecal incontinence. Patients were considered incontinent if they developed postsurgical incontinence to gas, liquid and/or solid stool, including soiling.

Study and patient characteristics collected included first author, study design, surgical procedure, number of patients and patient demographics, previous perianal surgeries including seton drainage, fistula aetiology, definition and percentage of success, recurrence (with and without transformation to an intersphincteric fistula after LIFT) and incontinence, and duration of follow‐up.

### Quality assessment

The methodological quality of the RCTs was assessed by the Cochrane Collaboration's tool for assessing risk of bias^13^. This tool focuses on selection bias, performance bias, detection bias, attrition bias, reporting bias and other bias, rated as low, high or unclear risk. For cohort studies, the Newcastle–Ottawa Quality Assessment Scale was used^14^. Stars were assigned in three different domains (selection, comparability and outcome), with a maximum total of nine stars. In the outcome domain, a minimum follow‐up period of 3 months and a maximum proportion of 5 per cent of subjects lost to follow‐up was considered acceptable. Studies were rated as good, fair or poor following the Agency for Healthcare Research and Quality (AHRQ) standard^15^, depending on the number of stars.

### Statistical analysis

Data analysis was done using OpenMeta[Analyst]^16^. A proportional meta‐analysis was performed comparing the overall success, recurrence and incontinence rates after the AF and LIFT procedures for cryptoglandular and Crohn's fistulas. Proportional meta‐analysis is an alternative approach in case of shortage or absence of RCTs^17^. Dichotomous data (overall success, recurrence and incontinence) were plotted using a random‐effects model, resulting in forest plots with pooled weighted proportions of the outcomes. A significant difference was defined as no overlap of the combined 95 per cent confidence intervals of the pooled proportions. Data were used from both RCTs and observational studies. Subgroup analyses were performed for overall success and recurrence rates in studies with a minimum follow‐up of 12 months. Another subgroup analysis was conducted for the recurrence rate after the LIFT procedure, to discriminate between recurrence of the initial tract and transformation to an intersphincteric tract. Heterogeneity was evaluated according to the *I* ^2^ statistic, and considered substantial when *I* ^2^ was 60 per cent or above. To assess the possibility of publication bias, funnel plots were created and evaluated for asymmetry.

## Results

### Study selection

**Figure 1:**
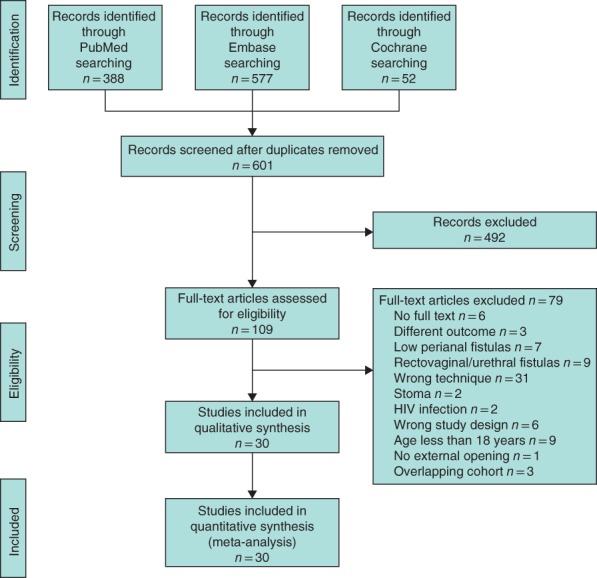
PRISMA flow chart for the review. HIV, human immunodeficiency virus

The initial literature search identified 1017 studies: 388 in PubMed, 577 in Embase and 52 from the Cochrane Library. After removal of duplicates 601 studies remained, and after screening of titles and abstracts 109 studies were selected for full‐text review. Following full‐text screening, 79 studies were excluded for various reasons (*Fig*. 1). Common reasons for exclusion were low perianal, rectovaginal or rectourethral fistulas, combinations or other variants of the techniques, and patients under the age of 18 years. The cohorts of three retrospective studies were overlapping. Thirty studies were finally included in the systematic review.

### Study and patient characteristics

Study and patient characteristics are shown in *Table* S1 (supporting information)^8^ ^9^, ^18^, ^19^, ^20^, ^21^, ^22^, ^23^, ^24^, ^25^, ^26^, ^27^, ^28^, ^29^, ^30^, ^31^, ^32^, ^33^, ^34^, ^35^, ^36^, ^37^, ^38^, ^39^, ^40^, ^41^, ^42^, ^43^, ^44^, ^45^. Five studies were RCTs, six were prospective cohort studies and 19 were retrospective cohort studies. Some 1548 patients were evaluated and a further 253 patients (with rectovaginal or low perianal fistulas) were excluded, leaving 1295 patients for pooled analyses (*Table* S2, supporting information). Of these patients, 1098 had fistulas of cryptoglandular origin, 64 had Crohn's fistulas and in 133 patients the underlying aetiology was unknown. The median number of patients included in the studies was 35 (range 5–252). The pooled percentage of women was 37·6 per cent and the range of median (or mean) ages was 32–52 years. Median (or mean) follow‐up ranged from 2 to 84 months. Of the 30 studies, two RCTs directly compared the AF and LIFT procedures, 16 investigated AF, and 12 investigated the LIFT procedure, frequently compared with another surgical or medical therapy. Three studies included fistulas of both cryptoglandular and Crohn's origin, 20 included fistulas of cryptoglandular origin, and three included fistulas of Crohn's origin. Unfortunately, fistula aetiology was not classified in four studies.

Definitions of success and recurrence were described in most studies, although inconsistencies were noted between the studies. For primary and overall success rates, all but three studies^18^, ^19^, ^20^ evaluated closure of the external fistula opening with or without the closure of the internal opening and intersphincteric wound after the LIFT procedure. The success rates of the other three studies^30^ ^31^, ^39^ were assessed by patient‐reported symptoms only. Recurrence of the initial fistula tract without transformation to an intersphincteric tract after the LIFT procedure was assessed in six studies^8^ ^31^, ^33^ ^34^, ^43^ ^44^. Other studies also scored intersphincteric wound recurrence, symptom recurrence, or provided no clear definition. Incontinence was measured inconsistently by patient‐reported symptoms or validated questionnaires (*Table* S2, supporting information).

### Risk‐of‐bias assessment

The overall methodological quality of the included RCTs was acceptable, with low risk of bias in most domains. Blinding measures, however, were not reported in some of the trials, resulting in an unclear risk of performance and detection bias (*Fig.* S1, supporting information). In contrast, only seven of the cohort studies were rated as good‐quality studies following the AHRQ standard^15^. The other 18 cohort studies were considered of poor quality, mostly due to lack of a control group (*Appendix* S2, supporting information).

### Overall success

#### Advancement flap

**Figure 2:**
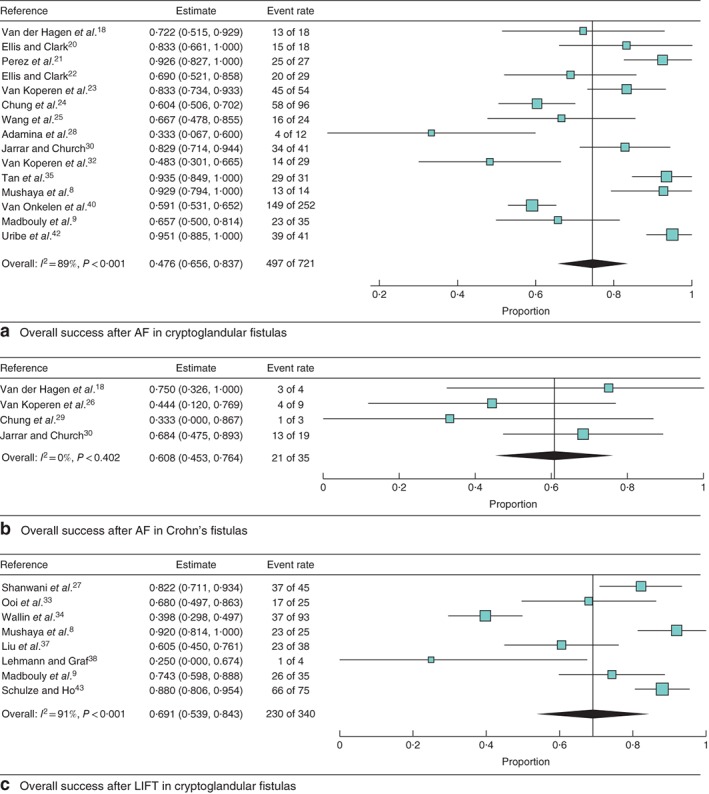
Forest plots of overall success of the two procedures in cryptoglandular and Crohn's fistula. **a** Advancement flap (AF) procedure in cryptoglandular fistula, **b** AF procedure in Crohn's disease and **c** ligation of intersphincteric fistula tract (LIFT) procedure in cryptoglandular fistula. A random‐effects model was used for meta‐analysis. Proportions are shown with 95 per cent confidence intervals

A pooled analysis for AF, not distinguishing for fistula aetiology, showed a weighted overall success rate of 69·9 (95 per cent c.i. 60·6 to 79·1) per cent (*I* ^2^ = 89·9 per cent) in 797 patients enrolled in 18 studies (*Fig.* S2, supporting information). Overall success rates ranged from 33·3 to 95·1 per cent across the studies, and median (or mean) duration of follow‐up varied widely (3–84 months). Subgroup analysis of 12 studies with a minimum follow‐up period of 12 months did not significantly change the weighted success rate (73·6 (62·6 to 84·7) per cent; *I* ^2^ = 90·8 per cent) (*Fig.* S3, supporting information). After separate analysis according to fistula aetiology, a weighted overall success rate of 74·6 (65·6 to 83·7) per cent (*I* ^2^ = 89·2 per cent) for cryptoglandular fistula and 61 (45 to 76) per cent) (*I* ^2^ = 0 per cent) for Crohn's perianal fistula was found (*Fig*. 2 *a,b*).

#### Ligation of intersphincteric fistula tract procedure

The pooled weighted overall success rate after LIFT was 68·9 (95 per cent c.i. 58·6 to 79·2) per cent (*I* ^2^ = 86·0 per cent), involving 488 patients from 13 different studies (*Fig.* S4, supporting information). Subgroup analysis of the eight studies with a minimum follow‐up of 12 months showed no significant change in the weighted success rate (64·9 (48·8 to 81·0) per cent; *I* ^2^ = 91·5 per cent) (*Fig.* S5, supporting information). The overall success rate after the LIFT procedure for cryptoglandular fistula was measured specifically in eight studies, and pooling the percentages resulted in a rate of 69·1 (53·9 to 84·3) per cent) (*I* ^2^ = 91·3 per cent) (*Fig*. 2 *c*). Only one study^45^, including 17 patients with a perianal fistula, reported the overall success rate in Crohn's perianal fistula after the LIFT procedure: 53 per cent after a median follow‐up of 23 months.

### Recurrence

#### Advancement flap

**Figure 3:**
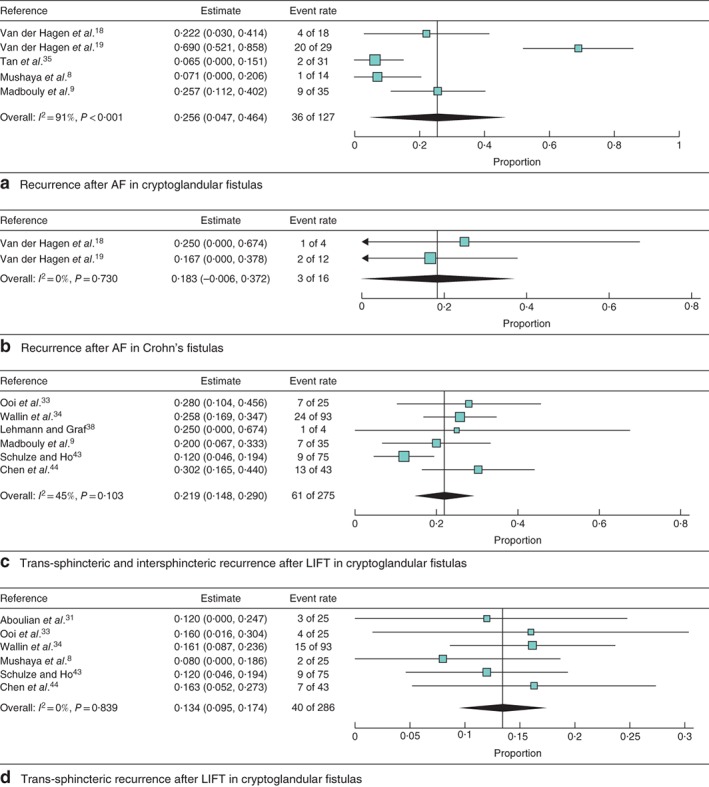
Forest plots of recurrence after the two procedures in cryptoglandular and Crohn's fistula. **a** Recurrence after advancement flap (AF) procedure in cryptoglandular fistula, **b** recurrence after AF procedure in Crohn's disease, **c** trans‐sphincteric and intersphincteric recurrence after ligation of intersphincteric fistula tract (LIFT) procedure in cryptoglandular fistula and **d** trans‐sphincteric recurrence after LIFT procedure in cryptoglandular fistula. A random‐effects model was used for meta‐analysis. Proportions are shown with 95 per cent confidence intervals

In total, five studies including 143 patients investigated the recurrence rate after the AF procedure. A pooled analysis, not distinguishing for fistula aetiology, showed a weighted recurrence rate of 22·6 (95 per cent c.i. 5·8 to 39·4) per cent (*I* ^2^ = 87·4 per cent) (*Fig.* S6, supporting information). Subgroup analysis of four studies with a minimum follow‐up period of 12 months showed a slightly, but not significantly, increased recurrence rate (27·2 (7·6 to 46·8) per cent; *I* ^2^ = 85·2 per cent) (*Fig.* S7, supporting information). For cryptoglandular fistula, the recurrence rate after AF was analysed in five studies, with a weighted recurrence rate of 25·6 (4·7 to 46·4) per cent (*I* ^2^ = 91·3 per cent) (*Fig*. 3 *a*). Only two retrospective studies with a total of 16 patients reported on recurrence rate in Crohn's perianal fistulas; pooling of the data resulted in a recurrence rate of 18 (0 to 37) per cent (*I* ^2^ = 0 per cent) (*Fig*. 3 *b*).

#### Ligation of intersphincteric fistula tract procedure

An overall weighted recurrence rate after the LIFT procedure of 21·9 (95 per cent c.i. 14·8 to 29·0) per cent (*I* ^2^ = 45·4 per cent) was observed in six studies including 275 patients (*Fig.* S8, supporting information). Subgroup analysis of the five studies with a minimum follow‐up of 12 months showed a similar weighted recurrence rate (21·2 (13·3 to 29·1) per cent; *I* ^2^ = 52·1 per cent) (*Fig.* S9, supporting information). Pooled analyses of six studies considering the LIFT procedure in cryptoglandular fistula showed a recurrence rate of 21·9 (14·8 to 29·0) per cent (*I* ^2^ = 45·4 per cent) (*Fig*. 3 *c*). Some fistulas transformed from a transsphincteric to an intersphincteric fistula; after exclusion of these recurrences the weighted recurrence rate decreased to 13·4 (9·5 to 17·4) per cent (*I* ^2^ = 0 per cent) (*Fig*. 3 *d*). No study reported the recurrence rate for Crohn's perianal fistula specifically after the LIFT procedure.

### Outcome analysis for incontinence

**Figure 4:**
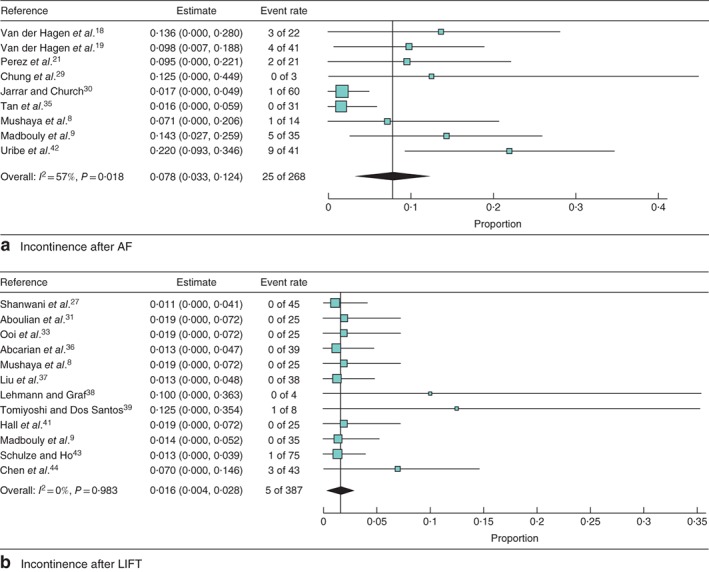
Forest plots of incontinence after the two procedures in cryptoglandular and Crohn's fistula combined. **a** Incontinence after advancement flap (AF) procedure and **b** incontinence after ligation of intersphincteric fistula tract (LIFT). A random‐effects model was used for meta‐analysis. Proportions are shown with 95 per cent confidence intervals

Rates of incontinence that developed after surgery were also pooled, but owing to small numbers no separate analyses for cryptoglandular or Crohn's fistula were performed. After the AF procedure (9 studies) the weighted incontinence rate was 7·8 (95 per cent c.i. 3·3 to 12·4) per cent (*I* ^2^ = 56·7 per cent) (*Fig*. 4 *a*). After the LIFT procedure (12 studies) a newly developed incontinence rate of 1·6 (0·4 to 2·8) per cent (*I* ^2^ = 0 per cent) was found (*Fig*. 4 *b*). The incontinence rate after the LIFT procedure was significantly lower than that following the AF procedure as the confidence intervals were not overlapping.

### Publication bias

The funnel plots (*Fig.* S10, supporting information) showed heterogeneity for all outcome measurements as not all studies were within the 95 per cent confidence interval. Asymmetry was found in the funnel plots for success after AF (missing some large positive studies), success after LIFT (missing some small positive studies) and incontinence after LIFT (missing some small negative studies). In general, the funnel plots had a broadly symmetrical distribution, indicating a low chance of publication bias.

## Discussion

This study suggests that for cryptoglandular fistula overall success and recurrence rates after the AF and LIFT procedure are comparable. In Crohn's fistula, the overall success rates after both procedures were also comparable, but data were limited and none of the studies reported recurrence rates after the LIFT procedure. Continence seems to be better preserved after the LIFT procedure for both indications, making the LIFT procedure an attractive surgical option for either disease.

The results observed after the AF procedure are in line with the systematic review of Soltani and Kaiser^46^, which showed weighted success and incontinence rates of 80·8 and 13·2 per cent respectively in cryptoglandular fistula, and 64·0 and 9·4 in Crohn's perianal fistula. The slightly different percentages observed in that study might be due to inclusion of additional procedures such as sphincteroplasty and defunctioning stomas, resulting in a more heterogeneous group of patients with perianal fistula. Results after the LIFT procedure were in some agreement with previous literature, as the systematic review of Yassin and colleagues^47^ showed a pooled success rate of 71 per cent for fistulas of multiple aetiologies. In the same systematic review^47^, minor disturbance in continence occurred in 6·0 per cent of the patients, but some of the studies did not correct for baseline incontinence.

The RCTs included in the present study were of reasonably good methodological quality, in contrast to most of the cohort studies, which were prone to bias. Owing to the small number of included RCTs with only two RCTs evaluating the LIFT procedure, the authors decided to pool the RCT and observational data. Pooling all available data and considering non‐overlapping 95 per cent confidence intervals as statistically significant is, however, unconventional. In general, it is preferable to analyse the different study designs separately, as outcomes of observational studies might be overestimated due to selection bias and confounding. In the absence of enough high‐quality studies, a proportional meta‐analysis is a valuable approach to summarize current knowledge. The heterogeneity in this meta‐analysis did not seem to be attributable to the different study designs, with comparable outcomes in the RCTs and cohort studies, and therefore a consistent point estimate after pooling of the data might be expected. The heterogeneity might be explained by differences in length of follow‐up, patient and fistula characteristics, and varying definitions of success, recurrence and incontinence. As the recurrence rate increases over time and late recurrences can be found 7–8 months after surgery^7^, separate meta‐analyses were performed for studies with a minimum follow‐up of 12 months. Adjustment for smoking, medication use, the number and length of fistula tracts, and duration of fistulizing disease was not possible. These factors might have affected the success and recurrence rates.

Inconsistencies in the definitions of success and recurrence might be one of the key factors influencing heterogeneity across the included studies. In an ideal world, postoperative outcomes would be confirmed by MRI as the sensitivity of physical examination alone is questionable^48^. Unfortunately, standardized radiological examination was performed in a minority of the present included studies. and in three studies outcomes were assessed by patient‐reported symptoms only. Although patient‐reported outcomes are important, especially in Crohn's fistula, where a Core Outcome Set has recently been devised that places them as the focus of the measurement of success^49^, an assessment of true fistula healing is also required to advance the science of fistula treatment towards producing treatments that eradicate fistulas in these patients. Length of follow‐up also varied considerably between studies, with some determining success after only a few months. Some fistula recurrence, particularly in Crohn's disease, occurs later than 1 year, so true healing rates of anal fistula can be determined only by long‐term follow‐up, or by MRI demonstrating deep tissue healing. Unfortunately, the definition of the latter is yet to be determined.

The instruments used to investigate incontinence varied widely, and measurement is hindered by the overlap with loss of faeces through a recurrent or persisting fistula. To assess the effect of the surgical procedure, the proportion of patients reporting newly developed incontinence to solids after surgery, as well as fluid, gas and soiling, was chosen as an endpoint. Nevertheless, a real estimation of continence preservation should be determined with preoperative and postoperative validated incontinence scores. Another limitation of this study is that discrimination between initial transsphincteric tract recurrence and recurrence in the intersphincteric wound after the LIFT procedure was lacking in some studies^50^. From a prognostic perspective, intersphincteric recurrence requires a less demanding treatment approach, as an intersphincteric fistula will have a high success rate after fistulotomy.

This study did not focus on some potential benefits of the LIFT procedure. It is technically simple and easy to perform, with possibly shorter operating times compared with AF^8^. Transformation to an intersphincteric fistula tract after the LIFT procedure reduces the complexity, with a higher chance of fistula closure in the future. However, AF remains important for non‐transsphincteric fistulas, fistulas with intersphincteric abscesses, fistulas with a high internal opening, or after unsuccessful closure by LIFT. In the subgroup of patients with Crohn's disease, surgical closure by AF can be attempted only in patients without proctitis or anorectal stenosis, and outcomes might improve under optimized medical therapy. Because of limited available data, future studies should investigate the applicability of the LIFT procedure in patients with active proctitis, and assess further its role in Crohn's perianal fistula, in particular in comparison with chronic seton drainage and biological therapy, as investigated in the PISA trial^51^.

## Supporting information


**Table S1** Study and patient characteristics
**Table S2** Fistula characteristics and definitions
**Appendix S1** Search terms
**Appendix S2** Risk of bias of cohort studies
**Fig. S1** Risk of bias summary of randomized controlled trials
**Fig. S2** Overall success after AF in total group (Crohn's and cryptoglandular fistulas)
**Fig. S3** Overall success after AF in total group (Crohn's and cryptoglandular fistulas) with minimal FU of 12 months
**Fig. S4** Overall success after LIFT in total group (Crohn's and cryptoglandular fistulas)
**Fig. S5** Overall success after LIFT in total group (Crohn's and cryptoglandular fistulas) with minimal FU of 12 months
**Fig. S6** Recurrence after AF in total group (Crohn's and cryptoglandular fistulas)
**Fig. S7** Recurrence after AF in total group (Crohn's and cryptoglandular fistulas) with minimal FU of 12 months
**Fig. S8** Recurrence after LIFT in total group (Crohn's and cryptoglandular fistulas)
**Fig. S9** Recurrence after LIFT in total group (Crohn's and cryptoglandular fistulas) with minimal FU of 12 months
**Fig. S10** Publication bias funnel plots
