# Supplementary material for: Systematic review and meta‐analysis of endorectal advancement flap and ligation of the intersphincteric fistula tract for cryptoglandular and Crohn's high perianal fistulas
Source: BJS Open. 2019 Jan 21;3(3):231–41. doi: 10.1002/bjs5.50129 (PMC6551488; doi:10.1002/bjs5.50129)
Supplement: Supplementary file 1 — Table S1 Study and patient characteristics Table S2 Fistula characteristics and definitions Appendix S1 Search terms Appendix S2 Risk of bias of cohort studies Fig. S1 Risk of bias summary of randomized controlled trials Fig. S2 Overall success after AF in total group (Crohn's and cryptoglandular fistulas) Fig. S3 Overall success after AF in total group (Crohn's and cryptoglandular fistulas) with minimal FU of 12 months Fig. S4 Overall success after LIFT in total group (Crohn's and cryptoglandular fistulas) Fig. S5 Overall success after LIFT in total group (Crohn's and cryptoglandular fistulas) with minimal FU of 12 months Fig. S6 Recurrence after AF in total group (Crohn's and cryptoglandular fistulas) Fig. S7 Recurrence after AF in total group (Crohn's and cryptoglandular fistulas) with minimal FU of 12 months Fig. S8 Recurrence after LIFT in total group (Crohn's and cryptoglandular fistulas) Fig. S9 Recurrence after LIFT in total group (Crohn's and cryptoglandular fistulas) with minimal FU of 12 months Fig. S10 Publication bias funnel plots [file BJS5-3-231-s001.docx]

**BJS5_50129**

**Systematic review and meta-analysis of endorectal advancement flap and ligation of the intersphincteric fistula tract for cryptoglandular and Crohn’s high perianal fistulas**

**M. E. Stellingwerf, E. M. van Praag, P. J. Tozer, W. A. Bemelman and C. J. Buskens**

**Table S1 Study and patient characteristics**

| **Author** | **Study design** | **Procedure** | **No. of patients** | **Median age in years** | **Sex, Female (%)** | **Seton (%)** | **Previous procedures** | **Median follow-up (mo)** |
| --- | --- | --- | --- | --- | --- | --- | --- | --- |
| **Van der Hagen *et al^18^*** | R | AF | 30 | 42 (22–68) | 15 (50) | 30 (100) | 26 (87) | 22 (8–52) |
| **Van der Hagen *et a1^19^*** | R | AF | 41 | 37 (23–72) | 15 (37) |  | 28 (68) | 72 (48–99) |
| **Ellis *et al^20^*** | RCT | AF | 30 | 47 (29-68) | 6 (20) |  |  | 22 (18 to 34) |
| **Perez *et al^21^*** | RCT | AF | 30 | 48 | 12 (44) | 0 (0) | 0 (0) | 38 (24–52) |
| **Ellis *et al^22^*** | R | AF | 68 | 38 (26-69) | 44 (65) |  |  | 10 |
| **Van Koperen *et al^23^*** | R | AF | 70 | 42 (21–67) | 23 (33) | 37 (39) | 29 (41) | 70 (22–127) |
| **Chung *et al^24^*** | R | AF | 96 | 46 (28-75) | 25 (26) |  |  | 3 |
| **Wang *et al^25^*** | R | AF | 26 | 39 (29-58) | 9 (35) | 16 (70) | 22 (85) | 27 (3–64) |
| **Van Koperen *et al^26^*** | R | AF | 9 | 36 (21–70) | 13 (59) | 3 (33) |  | 82 (17–132) |
| **Shanwani *et al^27^*** | P | LIFT | 45 | 42 ( 27–56) | 13 (29) | 0 (0) | 5 (11) | 9 (2–16) |
| **Adamina *et al^28^*** | R | AF | 12 | Mean 47 | 4 (33) | 12 (100) |  | 4 (1–40) |
| **Chung *et al^29^*** | R | AF | 5 | 32 (21–40) | 3 (60) | 0 (0) | 0 (0) | 3 |
| **Jarrar *et al^30^*** | R | AF | 75 | Mean 53 ± 14 | 55 (56) | 75 (100) |  | Mean 84 (21) |
| **Aboulian *et al^31^*** | R | LIFT | 25 | 39 | 7 (32) | 17 (65) | 7 (27) | 6 (2–13) |
| **Van Koperen *et al^32^*** | RCT | AF | 29 | 42 (24–61) | 10 (35) | 9 (31) | 20 (69) | 11 (5–27) |
| **Ooi *et al^33^*** | P | LIFT | 27 | 40 (21–67) | 8 (32) |  | 10 (40) | 6 (1–11) |
| **Wallin *et al^34^*** | R | LIFT | 112 | 43 (21–76) | 36 (39) | 70 (92) | 30 (32) | 19 (4–55) |
| **Tan *et al^35^*** | R | AF | 31 | 49 (19–74) | 4 (13) | 31 (100) |  | 6 (2–26) |
| **Abcarian *et al^36^*** | P | LIFT | 40 | 43 (22–70) |  |  | 29 (74) | 5 (1–16) |
| **Mushaya *et al^8^*** | RCT | LIFT | 25 | 48 (25 – 70) | 8 (32) | 25 (100) |  | 16 (IQR, 8–31) |
|  |  | AF | 14 | 48 (21 – 73) | 4 (29) | 14 (100) | 7 (18) | 30 (IQR, 6–35) |
| **Liu *et al^37^*** | R | LIFT | 38 | 42 (26–58) | 10 (26) | 29 (76) | 17 (100) | 26 (3–44) |
| **Lehmann *et al^38^*** | R | LIFT | 17 | 49 (30–76) | 8 (47) | 4 (24) |  | 14 (8–26) |
| **Tomiyoshi *et al^39^*** | P | LIFT | 8 | Mean 43 ( 21-68) | 6 (75) | 8 (100) | 0 (0) | 2 |
| **Van Onkelen *et al^40^*** | R | AF | 252 | 103 <45y, 149 ≥45y | 72 (29) | 38 (15) | 148 (59) | 21 (6–136) |
| **Hall *et al^41^*** | P | LIFT | 43 | Mean 45 ± 14 | 82 (34) |  |  | 3 |
| **Madbouly *et al^9^*** | RCT | LIFT  AF | 35  35 | Mean 45 ± 11  Mean 41 ± 12 | 16 (46)  12 (34) |  | 6 (17)  7 (20) | 12 |
| **Uribe *et al^42^*** | R | AF | 139 | Mean 52 ± 12 | 15 (37) |  |  | 23 |
| **Schulze *et al^43^*** | P | LIFT | 75 | Mean 50 (SE 1.4) | 24 (32) | 75 (100) | 36 (48) | Mean 15 (SE 2) |
| **Chen *et al^44^*** | R | LIFT | 43 | 37 (21–62) | 11 (26) |  | 12 (28) | 26 (13–63) |
| **Kaminski *et al^45^*** | R | LIFT | 23 | 39 (27-70) | 12 (52) | 16 (70) | 6 (26) | 23 (3–50) |

*RCT: Randomised controlled trial; P: Prospective cohort study; R: Retrospective cohort study
The values shown are median (range) or mean (SD)*

**Table S2 Fistula characteristics and definitions**

| **Author** | **Procedure** | **Excluded from analysis** | **Total analysed** | **Definition of success** | **Definition of recurrence (including intersphincteric recurrence)** | **Definition of recurrence (excluding intersphincteric recurrence)** | **Incontinence scores** |
| --- | --- | --- | --- | --- | --- | --- | --- |
| **Van der Hagen *et al^18^*** | AF | 8 | 22 | No drainage of the previous external opening with and without finger compression and no symptoms. | Recurrence after healing, confirmed by MRI. |  | Patient reported. |
| **Van der Hagen *et a1^19^*** | AF |  | 41 | No symptoms and no drainage of the previous external fistula opening with and without finger compression. | Patient reported recurrent symptoms. A different external opening and the same internal opening were interpreted as the same localization. |  | Patient reported. |
| **Ellis *et al^20^*** | AF | 12 | 18 |  |  |  |  |
| **Perez *et al^21^*** | AF | 3 | 27 |  |  |  | WCGS |
| **Ellis *et al^22^*** | AF | 39 | 29 |  |  |  |  |
| **Van Koperen *et al^23^*** | AF | 16 | 54 | Closed external opening and no experience of discharge or pain. |  |  | Vaizey and COREFO |
| **Chung *et al^24^*** | AF |  | 96 | Closure of the external fistula opening with no drainage or infection at 12 weeks postoperatively. |  |  |  |
| **Wang *et al^25^*** | AF | 2 | 24 | No drainage from the external opening. |  |  |  |
| **Van Koperen *et al^26^*** | AF |  | 9 | Closed external opening and no experience of discharge or pain. |  |  | Vaizey and COREFO |
| **Shanwani *et al^27^*** | LIFT |  | 45 | Complete epithelialization of the wound. |  |  | No fecal incontinence reported/ observed. |
| **Adamina *et al^28^*** | AF |  | 12 | The absence of drainage, abscess formation, and fistula openings on physical examination. |  |  |  |
| **Chung *et al^29^*** | AF | 2 | 3 | Closure of the external fistula opening with no drainage or infection. |  |  | Vaizey |
| **Jarrar *et al^30^*** | AF | 15 | 60 | No drainage after 6 weeks following. |  |  | Questionnaire of incontinence. |
| **Aboulian *et al^31^*** | LIFT |  | 25 | No persisting or recurrent symptoms. | Reopening of the external opening or conversion to intersphincteric tract. | Reopening of the external opening. | Patient reported. |
| **Van Koperen *et al^32^*** | AF |  | 29 | Closure of external and the internal openings and no discharge and pain. |  |  | COREFO, WIS and Vaizey |
| **Ooi *et al^33^*** | LIFT | 2 | 25 | Wound healing without recurrence. | Recurrence of initial tract including inner wound recurrence*.* | Recurrence of initial tract (not inner wound) | WIS and patient reported. |
| **Wallin *et al^34^*** | LIFT | 19 | 93 | Closure and absence of drainage or air leakage from the external opening or intersphincteric incision. | Recurrent air leakage or drainage from the external opening (or intersphincteric incision). | Recurrent air leakage or drainage from the external opening. | CCFI |
| **Tan *et al^35^*** | AF |  | 31 | Complete healing of the surgical wounds and the external opening. | Presence of recurrent discharge through the external opening. |  | Patient reported. |
| **Abcarian *et al^36^*** | LIFT |  | 40 | Absence of drainage from the external opening and intersphincteric incision. |  |  | Patient reported. |
| **Mushaya *et al^8^*** | LIFT |  | 25  14 | No recurrent fistula |  | Through the original tract and remained trans-sphincteric. | CCFI |
|  | AF |  |  |  |  |  |  |
| **Liu *et al^37^*** | LIFT |  | 38 | Healing of both the external opening and intersphincteric incision and no symptoms. |  |  | Patient reported. |
| **Lehmann *et al^38^*** | LIFT | 13 | 4 | No symptoms, complete healing of the external and internal orifices and the absence of gas or liquid in the fistula track. | Recurrence after apparent successful LIFT. |  | Patient reported de novo incontinence disturbance. |
| **Tomiyoshi *et al^39^*** | LIFT |  | 8 | Patients who denied leaking stool by wound. |  |  | De novo obvious injury of sphincter function. |
| **Van Onkelen *et al^40^*** | AF |  | 252 | Complete wound healing and closure of all external openings in combination with absence of symptoms. |  |  |  |
| **Hall *et al^41^*** | LIFT | 18 | 25 | The absence of a demonstrable fistula tract on physical examination without drainage. |  |  | CCFI |
| **Madbouly *et al^9^*** | LIFT  AF |  | 35  35 | Closed external and internal openings and no discharge. | Completely healed at any point followed by redischarge. |  | WCGS |
|  |  |  |  |  |  |  |  |
| **Uribe *et al^42^*** | AF | 98 | 41 | Complete wound healing and closure of all external openings and no symptoms. |  |  | WCGS and anorectal manometry |
| **Schulze *et al^43^*** | LIFT |  | 75 | Healing of the external opening and intersphincteric incision and no symptoms. | A downstaged tract from transsphincteric to intersphincteric fistula or a complete failure with the recurrent fistula tract extending from internal to external opening after initial healing. | A complete failure with the recurrent fistula tract extending from internal to external opening after initial healing. | CCFI |
| **Chen *et al^44^*** | LIFT |  | 43 | Closure and absence of drainage or air leakage from the external opening or intersphincteric space for at least more than 1 year. | Tract from the internal  opening to the external opening, with/without involvement of the intersphincteric wound. | Tract from the internal  opening to the external opening. | CCFI |
| **Kaminski *et al^45^*** | LIFT | 6 | 17 | Healing of the intersphincteric wound and closure of the external opening without anal pain. | Recurrence after apparent healing. |  |  |

WCGS: Wexner Continence Grading Scale (WCGS); COREFO: colorectal functional outcome; WIS: Wexner’s Incontinence Scale; CCFI: Cleveland Clinic Florida Fecal Incontinence score

**Appendix S1 Search terms**

**MEDLINE (PubMed)**

(Ligation[Mesh] OR ligation of intersphincteric fistula tract[tiab] OR LIFT[tiab] OR advancement*[tiab]) AND (“rectal fistula”[Mesh] OR anal fistul*[tiab] OR anorectal fistul*[tiab] OR perianal fistul*[tiab] OR fistula-in-ano[tiab])

**EMBASE (Ovid)**

1. ligation\
2. interphincteric fistula tract.mp.
3. LIFT.mp.
4. advancement*.mp.
5. 1 or 2 or 3 or 4
6. anus fistulas\
7. rectum fistula\
8. anorectal fistul*.mp.
9. fistula-in-ano.mp.
10. perianal fistul*.mp.
11. 6 or 7 or 8 or 9 or 10
12. 5 and 11

**The Cochrane Library**

1. MeSH descriptor: [Ligation] explode all trees
2. LIFT:ti,ab,kw (Word variations have been searched)
3. advancement:ti,ab,kw (Word variations have been searched)
4. ligation of intersphincteric fistula tract:ti,ab,kw (Word variations have been searched)
5. MeSH descriptor: [Rectal Fistula] explode all trees
6. anal fistula:ti,ab,kw (Word variations have been searched)
7. anorectal fistula:ti,ab,kw (Word variations have been searched)
8. perianal fistula:ti,ab,kw (Word variations have been searched)
9. fistula-in-ano:ti,ab,kw (Word variations have been searched)
10. #1 or #2 or #3 or #4
11. #5 or #6 or #7 or #8 or #9
12. #10 and #11

**Fig. S1 Risk of bias summary of randomized controlled trials**

|  | Random sequence generation (Selection bias) | Allocation concealment (Selection bias) | Blinding of participants and personnel (Performance bias) | Blinding of outcome assessment (Detection bias) | Incomplete outcome data (Attrition bias) | Selective reporting (Reporting bias) | Other bias |
| --- | --- | --- | --- | --- | --- | --- | --- |
| Ellis 2006^20^ |  |  |  |  |  |  |  |
| Madbouly 2014^9^ |  |  |  |  |  |  |  |
| Mushaya 2012^8^ |  |  |  |  |  |  |  |
| Perez 2006^21^ |  |  |  |  |  |  |  |
| Van Koperen 2011^32^ |  |  |  |  |  |  |  |

= High risk of bias

= Low risk of bias

= Unclear risk of bias

| **Assessment of quality of a cohort study**  **Newcastle–Ottawa Scale** | Prospective Cohort Studies | | | | | |
| --- | --- | --- | --- | --- | --- | --- |
| **Selection** (tick one box in each section) | Abcarian et al ^36^ | Ooi et al ^33^ | Hall et al ^41^ | Schulze et al ^43^ | Shanwani et al ^27^ | Tomiyoshi et al ^39^ |
| 1) Representativeness of the exposed cohort  a) truly representative of the average in the community ^★^  b) somewhat representative of the average in the community ^★^  c) selected group of users  d) no description of the derivation of the cohort | **★**  **🞏**  **🞏**  **🞏** | **🞏**  **★**  **🞏**  **🞏** | **★**  **🞏**  **🞏**  **🞏** | **★**  **🞏**  **🞏**  **🞏** | **🞏**  **★**  **🞏**  **🞏** | **🞏**  **★**  **🞏**  **🞏** |
| 2) Selection of the non exposed cohort  a) drawn from the same community as the exposed cohort ^★^  b) drawn from a different source  c) no description of the derivation of the non exposed cohort | **🞏**  **🞏**  **🗹** | **🞏**  **🞏**  **🗹** | **★**  **🞏**  **🞏** | **🞏**  **🞏**  **🗹** | **🞏**  **🞏**  **🗹** | **🞏**  **🞏**  **🗹** |
| 3) Ascertainment of exposure  a) secure record ^★^  b) structured interview ^★^  c) written self report  d) no description | **★**  **🞏**  **🞏**  **🞏** | **★**  **🞏**  **🞏**  **🞏** | **★**  **🞏**  **🞏**  **🞏** | **★**  **🞏**  **🞏**  **🞏** | **★**  **🞏**  **🞏**  **🞏** | **★**  **🞏**  **🞏**  **🞏** |
| 4) Demonstration that outcome of interest was not present at start of study  a) yes ^★^  b) no | **★**  **🞏** | **★**  **🞏** | **★**  **🞏** | **★**  **🞏** | **★**  **🞏** | **★**  **🞏** |

**Appendix S2 Risk of bias of cohort studies**

| **Assessment of quality of a cohort study**  **Newcastle–Ottawa Scale** | Prospective Cohort Studies | | | | | |
| --- | --- | --- | --- | --- | --- | --- |
| **Comparability** (tick none, one or both boxes, as appropriate) | Abcarian et al ^36^ | Ooi et al ^33^ | Hall et al ^41^ | Schulze et al ^43^ | Shanwani et al ^27^ | Tomiyoshi et al ^39^ |
| 1) Comparability of cohorts on the basis of the design or analysis  a) study controls for most important factor influencing the outcome (e.g. age, sex, previous studies etc.) ^★^  b) study controls for any additional factor ^★^ | **🞏**  **🞏** | **🞏**  **🞏** | **★**  **★** | **🞏**  **🞏** | **🞏**  **🞏** | **🞏**  **🞏** |
| **Outcome** (tick one box in each section) |  |  |  |  |  |  |
| 1) Assessment of outcome  a) independent blind assessment ^★^  b) record linkage ^★^  c) self report  d) no description | **🞏**  **★**  **🞏**  **🞏** | **🞏**  **★**  **🞏**  **🞏** | **🞏**  **★**  **🞏**  **🞏** | **🞏**  **★**  **🞏**  **🞏** | **🞏**  **★**  **🞏**  **🞏** | **🞏**  **★**  **🞏**  **🞏** |
| 2) Was follow-up long enough for outcomes to occur  a) yes, if median duration ≥ 3 months ^★^  b) no, if median duration < 3 months | **★**  **🞏** | **★**  **🞏** | **★**  **🞏** | **★**  **🞏** | **★**  **🞏** | **🞏**  **🗹** |
| 3) Adequacy of follow-up of cohorts  a) complete follow-up - all subjects accounted for ^★^  b) subjects lost to follow-up unlikely to introduce bias - small number lost - ≤ 5 % follow-up ^★^  c) follow-up rate < 95%  d) no statement | **🞏**  **★**  **🞏**  **🞏** | **★**  **🞏**  **🞏**  **🞏** | **★**  **🞏**  **🞏**  **🞏** | **★**  **🞏**  **🞏**  **🞏** | **★**  **🞏**  **🞏**  **🞏** | **★**  **🞏**  **🞏**  **🞏** |

| **Assessment of quality of a cohort study**  **Newcastle–Ottawa Scale** | Retrospective Cohort Studies | | | | | | | | |
| --- | --- | --- | --- | --- | --- | --- | --- | --- | --- |
| **Selection** (tick one box in each section) | Chung *et al* ^24^ | Ellis *et al* ^22^ | Jarrar *et al* ^30^ | Lehmann  *et al* ^38^ | Aboulian  *et al* ^31^ | Liu *et al* ^37^ | Tan *et al* ^35^ | Uribe *et al* ^42^ | Van der Hagen *et al* ^18^ |
| 1) Representativeness of the exposed cohort  a) truly representative of the average in the community ^★^  b) somewhat representative of the average in the community ^★^  c) selected group of users  d) no description of the derivation of the cohort | **🞏**  **★**  **🞏**  **🞏** | **🞏**  **★**  **🞏**  **🞏** | **🞏**  **★**  **🞏**  **🞏** | **🞏**  **★**  **🞏**  **🞏** | **🞏**  **★**  **🞏**  **🞏** | **🞏**  **★**  **🞏**  **🞏** | **🞏**  **★**  **🞏**  **🞏** | **🞏**  **★**  **🞏**  **🞏** | **🞏**  **★**  **🞏**  **🞏** |
| 2) Selection of the non-exposed cohort  a) drawn from the same community as the exposed cohort ^★^  b) drawn from a different source  c) no description of the derivation of the non exposed cohort | **★**  **🞏**  **🞏** | **★**  **🞏**  **🞏** | **🞏**  **🞏**  **🗹** | **🞏**  **🞏**  **🗹** | **🞏**  **🞏**  **🗹** | **🞏**  **🞏**  **🗹** | **★**  **🞏**  **🞏** | **★**  **🞏**  **🞏** | **🞏**  **🞏**  **🗹** |
| 3) Ascertainment of exposure  a) secure record ^★^  b) structured interview ^★^  c) written self report  d) no description | **★**  **🞏**  **🞏**  **🞏** | **★**  **🞏**  **🞏**  **🞏** | **★**  **🞏**  **🞏**  **🞏** | **★**  **🞏**  **🞏**  **🞏** | **★**  **🞏**  **🞏**  **🞏** | **★**  **🞏**  **🞏**  **🞏** | **★**  **🞏**  **🞏**  **🞏** | **★**  **🞏**  **🞏**  **🞏** | **★**  **🞏**  **🞏**  **🞏** |
| 4) Demonstration that outcome of interest was not present at start of study  a) yes ^★^  b) no | **★**  **🞏** | **★**  **🞏** | **★**  **🞏** | **★**  **🞏** | **★**  **🞏** | **★**  **🞏** | **★**  **🞏** | **★**  **🞏** | **★**  **🞏** |

| **Assessment of quality of a cohort study**  **Newcastle–Ottawa Scale** | | Retrospective Cohort Studies | | | | | | | | | | | | | | | | | |  |
| --- | --- | --- | --- | --- | --- | --- | --- | --- | --- | --- | --- | --- | --- | --- | --- | --- | --- | --- | --- | --- |
| **Comparability** (tick none, one or both boxes, as appropriate) | | Chung  *et al* ^24^ | | Ellis *et al* ^22^ | | Jarrar  *et al* ^30^ | | Lehmann  *et al* ^38^ | | Aboulian  *et al* ^31^ | | Liu *et al* ^37^ | | Tan *et al* ^35^ | | Uribe *et al* ^42^ | | Van der Hagen  *et al* ^18^ | |  |
| 1) Comparability of cohorts on the basis of the design or analysis  a) study controls for most important factor influencing the outcome (e.g. age, sex, previous studies etc.) ^★^  b) study controls for any additional factor ^★^ | | **★**  **🞏** | | **★**  **★** | | **🞏**  **🞏** | | **🞏**  **🞏** | | **🞏**  **🞏** | | **🞏**  **🞏** | | **🞏**  **🞏** | | **🞏**  **🞏** | | **🞏**  **🞏** | |  |
| **Outcome** (tick one box in each section) | |  | |  | |  | |  | |  | |  | |  | |  | |  | |  |
| 1) Assessment of outcome  a) independent blind assessment ^★^  b) record linkage ^★^  c) self report  d) no description | | **🞏**  **★**  **🞏**  **🞏** | | **🞏**  **★**  **🞏**  **🞏** | | **🞏**  **🞏**  **🗹**  **🞏** | | **🞏**  **★**  **🞏**  **🞏** | | **🞏**  **★**  **🞏**  **🞏** | | **🞏**  **★**  **🞏**  **🞏** | | **🞏**  **★**  **🞏**  **🞏** | | **🞏**  **★**  **🞏**  **🞏** | | **🞏**  **★**  **🞏**  **🞏** | |  |
| 2) Was follow-up long enough for outcomes to occur  a) yes, if median duration ≥ 3 months ^★^  b) no, if median duration < 3 months | | **★**  **🞏** | | **★**  **🞏** | | **★**  **🞏** | | **★**  **🞏** | | **★**  **🞏** | | **★**  **🞏** | | **★**  **🞏** | | **★**  **🞏** | | **★**  **🞏** | |  |
| 3) Adequacy of follow-up of cohorts  a) complete follow-up - all subjects accounted for ^★^  b) subjects lost to follow-up unlikely to introduce bias - small number lost - ≤ 5 % follow-up ^★^  c) follow-up rate < 95%  d) no statement | | **★**  **🞏**  **🞏**  **🞏** | | **★**  **🞏**  **🞏**  **🞏** | | **🞏**  **🞏**  **🗹**  **🞏** | | **🞏**  **🞏**  **🗹**  **🞏** | | **★**  **🞏**  **🞏**  **🞏** | | **★**  **🞏**  **🞏**  **🞏** | | **★**  **🞏**  **🞏**  **🞏** | | **★**  **🞏**  **🞏**  **🞏** | | **★**  **🞏**  **🞏**  **🞏** | |  |
| **Assessment of quality of a cohort study**  **Newcastle–Ottawa Scale** | Retrospective Cohort Studies | | | | | | | | | | | | | | | | | | | |
| **Selection** (tick one box in each section) | Van der Hagen *et al* ^19^ | | Van Koperen *et al* ^23^ | | Van Onkelen *et al* ^40^ | | Wallin *et al* ^34^ | | Wang *et al* ^25^ | | Van Koperen *et al* ^26^ | | Chung *et al* ^29^ | | Adamina *et al* ^28^ | | Chen *et al ^44^* | | Kaminski *et al ^45^* | |
| 1) Representativeness of the exposed cohort  a) truly representative of the average in the community ^★^  b) somewhat representative of the average in the community ^★^  c) selected group of users  d) no description of the derivation of the cohort | **🞏**  **★**  **🞏**  **🞏** | | **🞏**  **★**  **🞏**  **🞏** | | **🞏**  **★**  **🞏**  **🞏** | | **★**  **🞏**  **🞏**  **🞏** | | **🞏**  **★**  **🞏**  **🞏** | | **🞏**  **★**  **🞏**  **🞏** | | **🞏**  **★**  **🞏**  **🞏** | | **🞏**  **★**  **🞏**  **🞏** | | **🞏**  **★**  **🞏**  **🞏** | | **🞏**  **★**  **🞏**  **🞏** | |
| 2) Selection of the non-exposed cohort  a) drawn from the same community as the exposed cohort ^★^  b) drawn from a different source  c) no description of the derivation of the non exposed cohort | **★**  **🞏**  **🞏** | | **★**  **🞏**  **🞏** | | **🞏**  **🞏**  **🗹** | | **🞏**  **🞏**  **🗹** | | **🞏**  **🗹**  **🞏** | | **★**  **🞏**  **🞏** | | **★**  **🞏**  **🞏** | | **🞏**  **🗹**  **🞏** | | **🞏**  **🞏**  **🗹** | | **🞏**  **🞏**  **🗹** | |
| 3) Ascertainment of exposure  a) secure record ^★^  b) structured interview ^★^  c) written self report  d) no description | **★**  **🞏**  **🞏**  **🞏** | | **🞏**  **★**  **🞏**  **🞏** | | **★**  **🞏**  **🞏**  **🞏** | | **★**  **🞏**  **🞏**  **🞏** | | **★**  **🞏**  **🞏**  **🞏** | | **★**  **🞏**  **🞏**  **🞏** | | **★**  **🞏**  **🞏**  **🞏** | | **★**  **🞏**  **🞏**  **🞏** | | **★**  **🞏**  **🞏**  **🞏** | | **🞏**  **★**  **🞏**  **🞏** | |
| 4) Demonstration that outcome of interest was not present at start of study  a) yes ^★^  b) no | **★**  **🞏** | | **★**  **🞏** | | **★**  **🞏** | | **★**  **🞏** | | **★**  **🞏** | | **★**  **🞏** | | **★**  **🞏** | | **★**  **🞏** | | **★**  **🞏** | | **★**  **🞏** | |

| **Assessment of quality of a cohort study**  **Newcastle–Ottawa Scale** | Retrospective Cohort Studies | | | | | | | | | |
| --- | --- | --- | --- | --- | --- | --- | --- | --- | --- | --- |
| **Comparability** (tick none, one or both boxes, as appropriate) | Van der Hagen *et al* ^19^ | Van Koperen *et al* ^23^ | Van Onkelen *et al* ^40^ | Wallin *et al* ^34^ | Wang *et al* ^25^ | Van Koperen *et al* ^26^ | Chung *et al* ^29^ | Adamina *et al* ^28^ | Chen *et al ^44^* | Kaminski *et al ^45^* |
| 1) Comparability of cohorts on the basis of the design or analysis  a) study controls for most important factor influencing the outcome (e.g. age, sex, previous studies etc.) ^★^  b) study controls for any additional factor ^★^ | **🞏**  **🞏** | **★**  **★** | **🞏**  **🞏** | **🞏**  **🞏** | **🞏**  **🞏** | **★**  **★** | **★**  **★** | **★**  **★** | **🞏**  **🞏** | **🞏**  **🞏** |
| **Outcome** (tick one box in each section) |  |  |  |  |  |  |  |  |  |  |
| 1) Assessment of outcome  a) independent blind assessment ^★^  b) record linkage ^★^  c) self report  d) no description | **🞏**  **★**  **🞏**  **🞏** | **🞏**  **★**  **🞏**  **🞏** | **★**  **🞏**  **🞏**  **🞏** | **🞏**  **🞏**  **🗹**  **🞏** | **🞏**  **🞏**  **🗹**  **🞏** | **🞏**  **★**  **🞏**  **🞏** | **🞏**  **★**  **🞏**  **🞏** | **🞏**  **★**  **🞏**  **🞏** | **🞏**  **🞏**  **🗹**  **🞏** | **🞏**  **★**  **🞏**  **🞏** |
| 2) Was follow-up long enough for outcomes to occur  a) yes, if median duration ≥ 3 months ^★^  b) no, if median duration < 3 months | **★**  **🞏** | **★**  **🞏** | **★**  **🞏** | **★**  **🞏** | **★**  **🞏** | **★**  **🞏** | **★**  **🞏** | **★**  **🞏** | **★**  **🞏** | **★**  **🞏** |
| 3) Adequacy of follow-up of cohorts  a) complete follow-up - all subjects accounted for ^★^  b) subjects lost to follow-up unlikely to introduce bias - small number lost - ≤ 5 % follow-up ^★^  c) follow-up rate < 95%  d) no statement | **🞏**  **🞏**  **🗹**  **🞏** | **🞏**  **🞏**  **🗹**  **🞏** | **★**  **🞏**  **🞏**  **🞏** | **🞏**  **🞏**  **🗹**  **🞏** | **★**  **🞏**  **🞏**  **🞏** | **🞏**  **🞏**  **🗹**  **🞏** | **★**  **🞏**  **🞏**  **🞏** | **★**  **🞏**  **🞏**  **🞏** | **★**  **🞏**  **🞏**  **🞏** | **★**  **🞏**  **🞏**  **🞏** |

**Fig. S2 Overall success after AF in total group (Crohn’s and cryptoglandular fistulas)

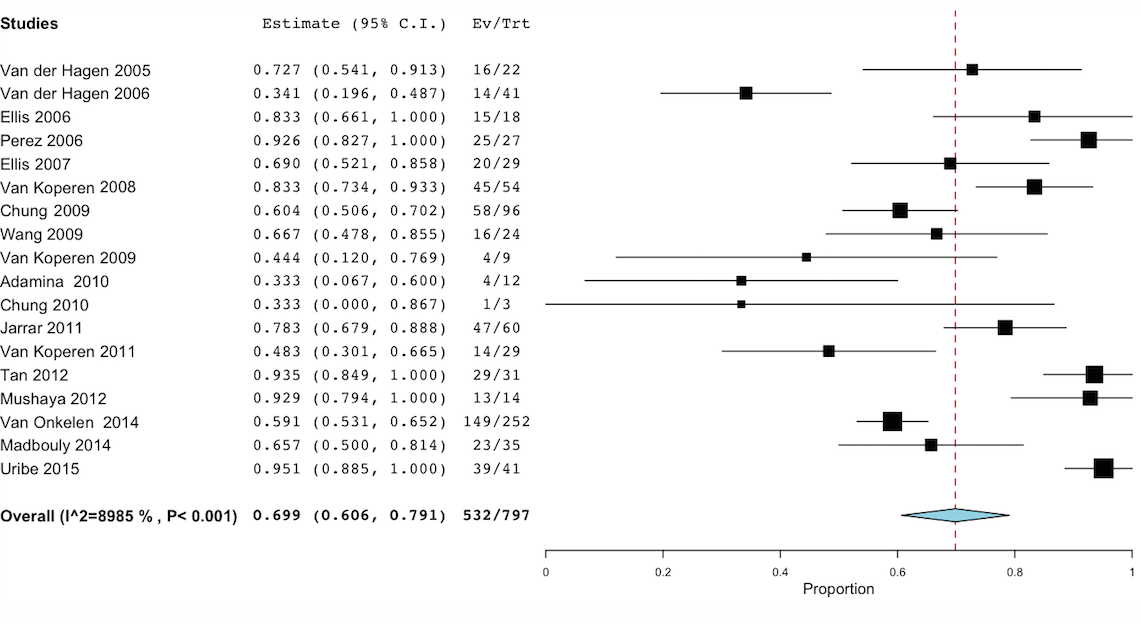
**

**Fig. S3 Overall success after AF in total group (Crohn’s and cryptoglandular fistulas) with minimal FU of 12 months


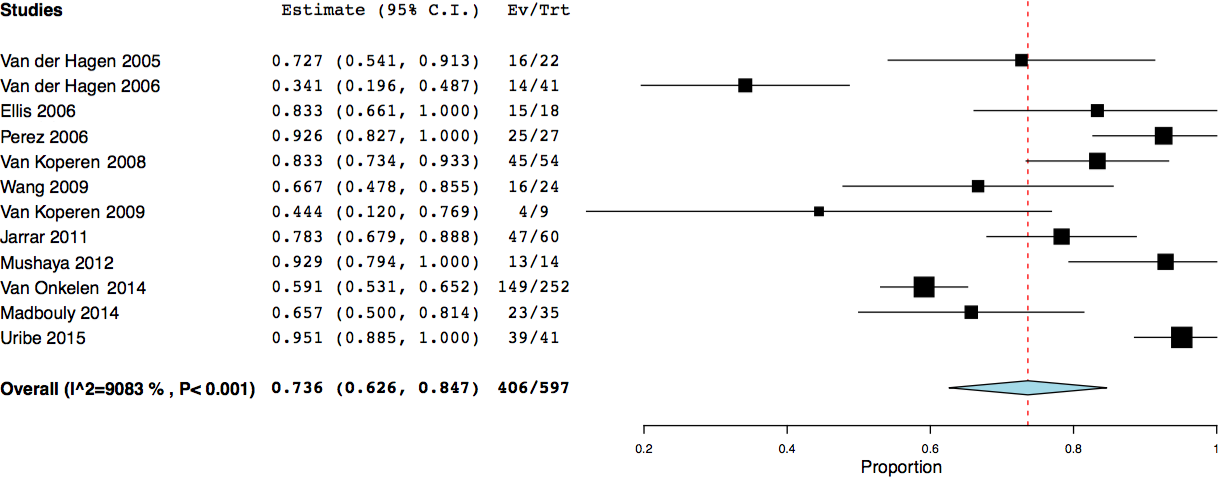
**

**Fig. S4 Overall success after LIFT in total group (Crohn’s and cryptoglandular fistulas)**

**
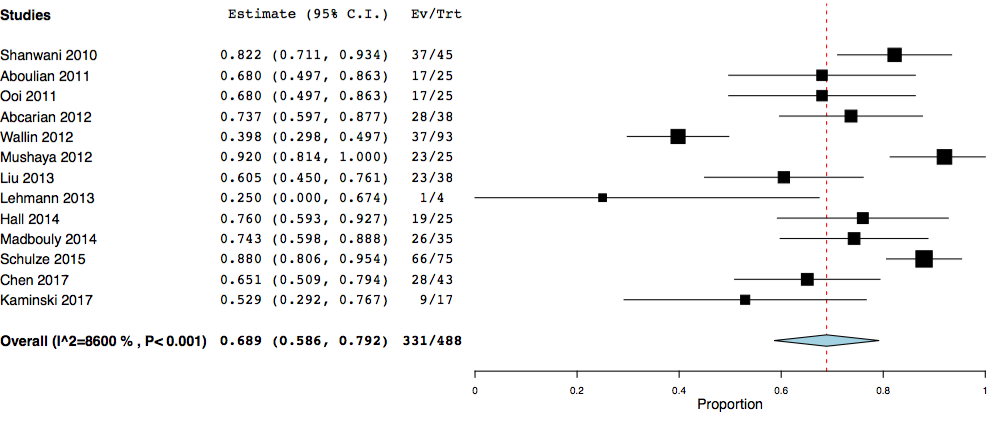
**

**Fig. S5 Overall success after LIFT in total group (Crohn’s and cryptoglandular fistulas) with minimal FU of 12 months


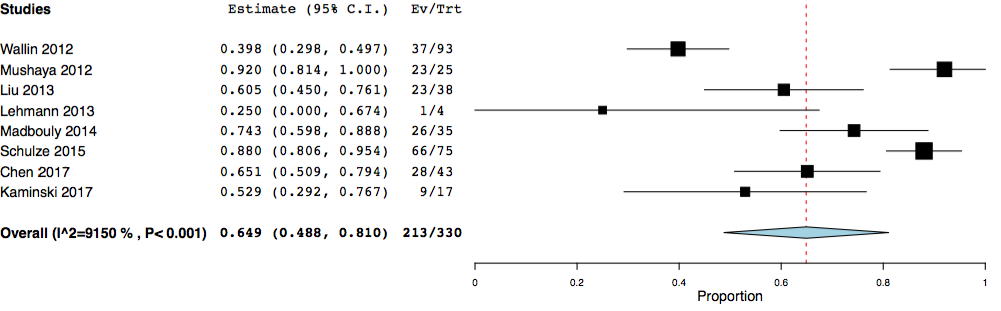
**

**Fig. S6 Recurrence after AF in total group (Crohn’s and cryptoglandular fistulas)

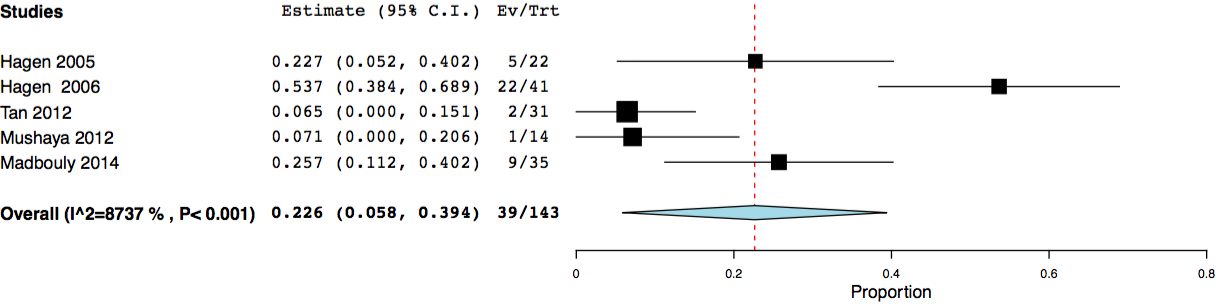
**

**Fig. S7 Recurrence after AF in total group (Crohn’s and cryptoglandular fistulas) with minimal FU of 12 months

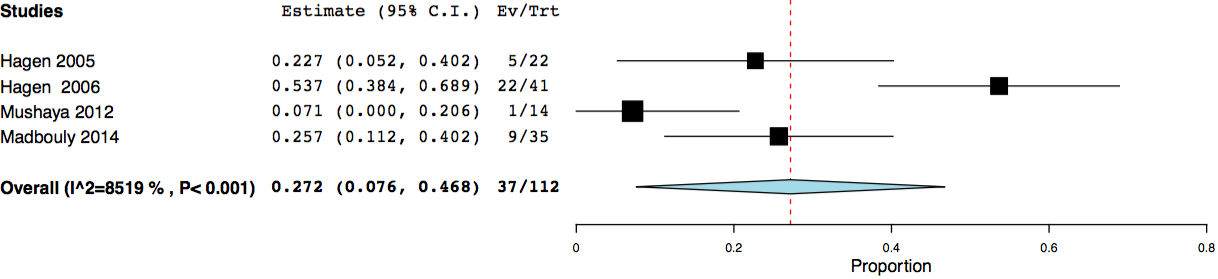
**

**Fig. S8 Recurrence after LIFT in total group (Crohn’s and cryptoglandular fistulas)


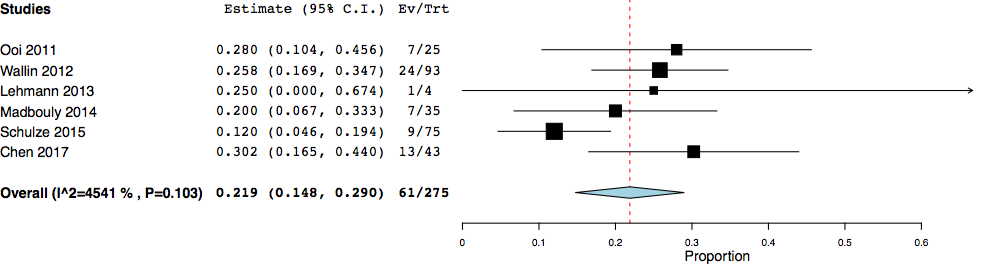
**

**Fig. S9 Recurrence after LIFT in total group (Crohn’s and cryptoglandular fistulas) with minimal FU of 12 months


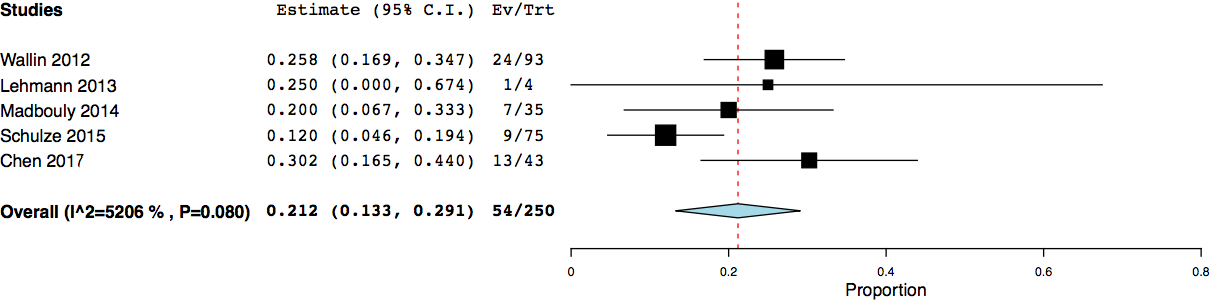
**

**Fig. S10 Publication bias funnel plots**

**
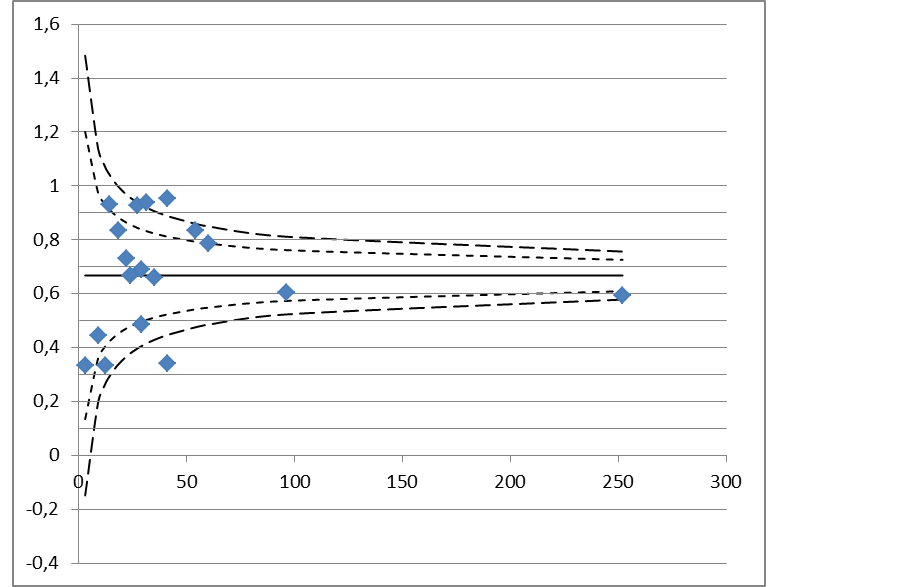

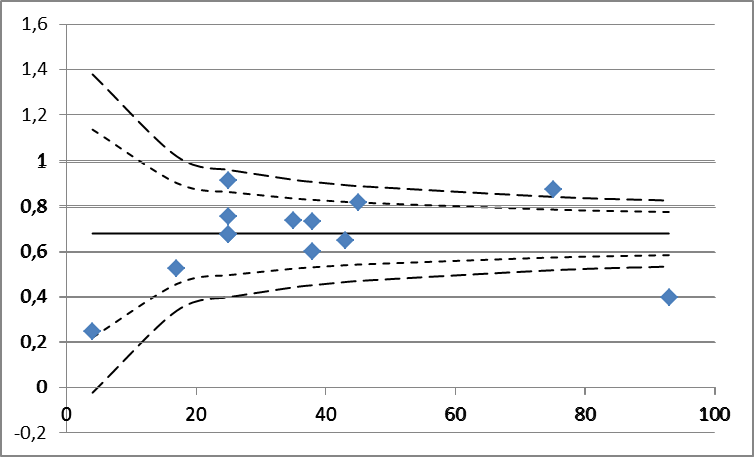

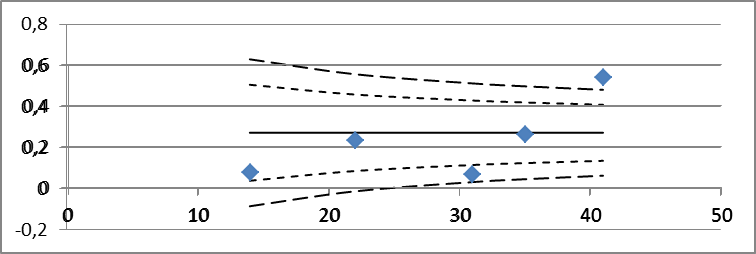

1.** Funnel plot success rate AF total **2.** Funnel plot success rate LIFT total **3.** Funnel plot recurrence rate AF total

**
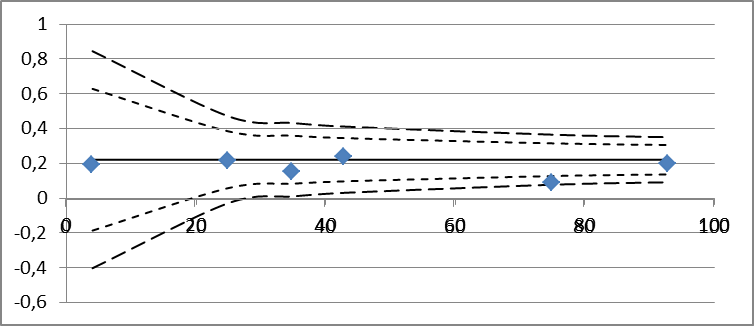

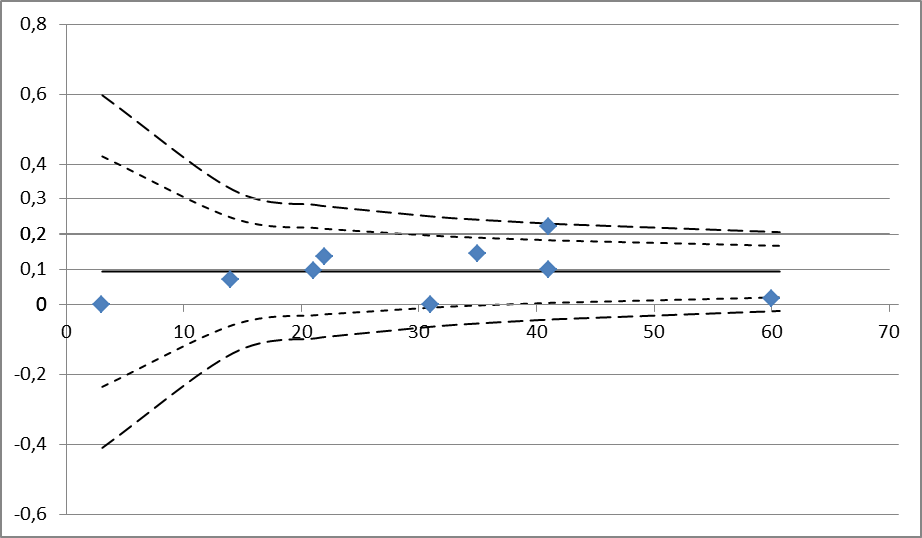

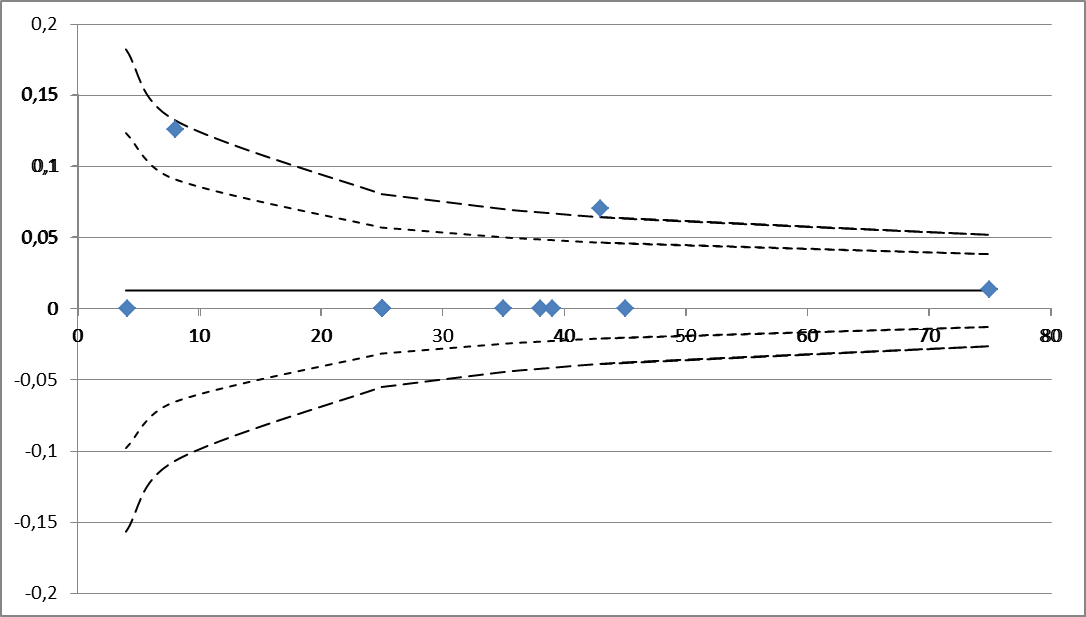

4.** Funnel plot recurrence rate LIFT total **5.** Funnel plot incontinence rate AF total **6.** Funnel plot incontinence rate LIFT total
